# Natural Immunomodulators 2018

**DOI:** 10.1155/2019/4341698

**Published:** 2019-11-03

**Authors:** Daniel Ortuño-Sahagún, Ajay K. S. Rawat, Kurt Zänker

**Affiliations:** ^1^Instituto de Investigación en Ciencias Biomédicas (IICB), CUCS, Universidad de Guadalajara, Guadalajara, JAL, Mexico; ^2^National Botanical Research Institute, Lucknow, Uttar Pradesh, India; ^3^Witten/Herdecke University, Witten, Germany

In 2017, the signed editors curated at the state of the art of immune science special research results which were performed with natural compounds and which approached immunomodulation at different molecular levels and for different diseases [1]. The original manuscripts (ms) were published in the prestigious *Journal of Immunology Research*, 2017, released by the Hindawi Publishing Corporation. This special issue was very successful, well taken by the readers, and therefore, there was an urgent demand by the scientific community to get updated latest news in the field of natural immunomodulators. Here, in 2019, we present 13 peer-reviewed manuscripts (ms) at the cutting edge of natural immunomodulators.

The concept of immunomodulation is, of course, not a new one. The science behind immunomodulation—in general—was carried out with vaccines and by vaccination and is closely connected, among others, to names like Edward Jenner, Louis Pasteur, Emil von Behring, and Jonas Salk. The biochemical preparations of antigens inducing immunogenicity and provoking a desired immune defense in the body were a milestone in fighting against infectious diseases—this was due to a Western paradigm of medicine.

However, in a unique and holistic world, there is another tale to give medical and philosophical advice to patients, called holistic medicine, which does not follow a reductionist approach, likewise to repair—often successfully—symptoms of a diseased body according to a machine model. The more we are learning that the immune system is navigating the biopsychosocial well-being and behavior of patients, the more we try to decipher biological and psychological factors which can influence any constituent and function of the immune system in a specific or nonspecific manner by targeting the innate and/or the adaptive arm of the immune response.

With these annual special issues, we make known integrative knowledge of immunomodulation, which started out in the past mainly by vaccination, but which will be in the future multifacetedly shaped by increasingly gathered knowledge about details of herbs and diet, traditional Chinese medicine (TCM), Ayurvedic medicine, psychological interventions, and many other treat-to-target approaches in order to manipulate the immune system for the benefit of the individual, focusing, necessarily in dissecting the cellular and molecular mechanisms subjacent to the immunomodulatory effects of natural compounds, and for the discovering of novel promising candidates that can be used in the future immunotherapeutic strategies.

One area of recent outstanding interest is the study of plant-derived immunomodulators [2]. Here, we included supporting evidence on the mechanism of action, as immunomodulators, of nutraceuticals obtained from *Curcuma longa* (curcumin), *Carthamus tinctorius* (safflower), and from *Alternanthera sessilis.*

Two ms by J. R. Macías-Perez et al. addressed the mode of action of curcumin with respect to hepatoprotection against amoebic liver abscess and as cotreatment to reverse liver cirrhosis in a hamster model. These results are of importance for the molecular understanding and treating of common liver diseases and illnesses, which have an enormous personal disease-driven impact and have social and financial influence on the public health system in countries with low income. The presented results propose that curcumin is a natural immunomodulator which can be used as a treat-to-target compound.

We accepted the ms by H. Liao et al. because the group lined out the importance of the different effects of safflower on protein and mRNA expression of iNOS and IL-1*β* caused by decoction and injection. The results have to be taken as a clear message that (i) any pharmacological preparation of botanicals should be critically scrutinized and (ii) the routing of administration of herbal immunomodulators has to be evaluated in clinical studies.

This continues with the ms by K. Muniandy et al. The group nicely demonstrated that a stem extract of *Alternanthera sessilis* targets the NF-*κ*B pathway and suppresses proinflammatory cytokine production including proinflammatory mediators like NO and PGE2. NF-*κ*B is one important, if not the key molecule regulating and controlling infections—but very Janus-faced; it is of high interest that such a herbal stem extract selectively suppresses the proinflammatory arm of the immune system by targeting NF-*κ*B.

In addition to plant compounds, there is a huge research on natural compounds able to modulate the immune function. Here, we include an excellent review of the effect of natural compounds on NK cell activation. M. Grudzien and A. Rapak describe constituents—from vitamins to polyphenols and botanical extracts—which are able to modify the NK activity. This summary might be a scientific proposal for colleagues to consider one of these constituents as natural immunomodulators by using them in different clinical settings.

Another area of great interest is the immune response to pathogens [3], as in the case of the manuscript by R. Zheng et al. which addressed the cellular T lymphocyte response to Brucella infections and demonstrated that T cell subsets are imbalanced, and the adaptive defense arm of the immune system is malfunctioning in patients affected by Brucellosis. Within the follow-up time of the clinical study, the patients did not completely recover, so the suggestion by the authors is to extend the convalescence period in further studies in order to understand over time increasingly and adequately the modified T cell subset answers. Of further attention is the response to LPS, presented by L. M. Kanevskiy et al. showing results on human NK cells when stimulated with LPS. It was a prevailing opinion that human NK cells do not almost express TLR4 receptors, which are required for LPS stimulation. However, the stimulation response of NK cells, e.g., by producing interferon-gamma, heavily depends on the O-antigen structure of the LPS. Moreover, the interferon-gamma production between NK cells and macrophages stimulated by mutant LPS compounds was compared and they found that NK cells can be significantly shifted to higher interferon-gamma production as seen under similar experimental conditions by macrophages.

The immunomodulatory properties of animal substances and immunotoxicology have attracted increasing attention [4]. Here, a couple of very interesting works deals with these aspects. The group of J. J. Mora Román et al. reports about the immunomodulating potential of mollusk hemocyanins (KLH) in combination with human vaccine adjuvants in a murine model for oral cancer. The results suggest that the use of KLH allows a dosage tapering of different adjuvants like alum, AddaVax, and QS-21. Moreover, the experimental *in vivo* results with transplanted MOC-7 cells further suggest that KLH do exhibit immunomodulation activities by itself. In addition, the ms from the group of G. Mellado-Sánchez et al. is pointing out that immunogenicity against a human dialyzable leucocyte extracts (hDLEs) can be generated by two strategies, (i) by chemical conjugation of peptides to a carrier protein as the immunogen and (ii) by using rabbits to induce antibodies against the peptides in general. These approaches demonstrate in general the impact of developing surrogate antibodies, which might be directed to any chosen target, as exemplified with the hDLEs.

Chemical compounds, as artificial sweeteners and vaccine adjuvants, are also targets of studies on their immunomodulatory properties. That is the case of the ms by A. Y. Gomez-Arauz et al. that is picking up a tremendous public health problem, which is of great worldwide importance—the metabolic syndrome—and therapeutic intervention options. The use of artificial sweeteners is very common, but almost nothing is known about their influence on the immune system. The results clearly show that innocuous consumption of sucralose has to be critically considered by caregivers with expertise in metabolic diseases (diabetes, obesity) because the expression patterns of monocytes (subpopulations) are enigmatically altered.

As mentioned, a ms by A. P. Jiménez-Uribe et al. about the molecular activities on the expression of cytokine levels induced by hDLEs, when used as coadjuvant, is published in this issue. We considered the results as important, because the authors show that the hDLE (Transferon) is a potent immunomodulator; the spectrum of which—according to immune modulation—has to be determined in further studies in order to demonstrate in more clinical safety detail.

Finally, several aspects of immunomodulatory therapies for clinical development of therapeutic are remarkable and essential to attend [5]. For instance, here, we present some immunomodulatory aspects related with cancer and with organ transplant. First, a review by J. Li et al. enlightened the evolving roles of macrophages in organ transplantation (OT). OT is a very successful and lifesaving strategy for patients with end-stage organ failure. It is one of the prime examples in modern Western medicine for success, and the publications about OT are multiplexed. Yet, we have selected this ms, because the authors nicely discuss the emerging roles of macrophages on organ transplantation, rejection, and transplant tolerance in a polytextural content, which is often missing in others, even highly ranked publications. Also, we accepted the ms by I. Martínez-Reza et al. because any news with respect to a progress in fighting against triple negative breast cancer (TNBC) are good news. The group nicely demonstrated that calcitriol has antiproliferative effects via molecular pathways including IL-1*β* and TNF-alpha and the receptors of these cytokines. For the future, calcitriol can be considered as immunomodulator in TNBC.

We all—the editors and authors with a high reputation in and passion for immunology—are fighting against different immune system-driven maladies and, hopefully, will be able to strengthen sufficiently the immune competence of immunocompromised patients to regain their health benefit. These distributed results are surely a good clinical basis and an evidential rationale to consider immunomodulators in clinical settings.
